# Data set for power system reliability analysis using a four-area test network

**DOI:** 10.1016/j.dib.2020.106495

**Published:** 2020-11-04

**Authors:** Iver Bakken Sperstad, Espen Hafstad Solvang, Sigurd Hofsmo Jakobsen, Oddbjørn Gjerde

**Affiliations:** SINTEF Energy Research, Trondheim, Norway

**Keywords:** Power system reliability, grid model, network model, power system model, reliability data, power market analysis

## Abstract

This article presents a test data set combining data relevant for power system reliability analysis, including network data, reliability data, basic interruption cost data, and exemplary operating state data. The data set originated as a data set for testing power market models with network constraints and was later extended for use in integrated power market and power system reliability analyses. The network model consists of 25 buses and four price (market) areas representing small regions of the Nordic power system. Three of the areas are meshed but with relatively weak connections between them, and a fourth area is represented by a single bus connected by an HVDC cable. Reliability data (failure rates and outage times) are based on statistics from the Norwegian standardised system FASIT for collection, calculation and reporting of disturbance and reliability data.

## Specifications Table

**Table:** 

Subject	Electrical and Electronic Engineering
Specific subject area	Power system reliability analysis
Type of data	Tables
How data were acquired	By combining power system component data, reliability data, interruption cost data and load demand data with a synthetically generated network model.
Data format	Secondary
Parameters for data collection	For reliability data: Based on historical data from 1983 to 2005. For interruption cost data: Based on the Norwegian cost of energy supply scheme valid from 2014. For other parts of the data set: Combined from different sources.
Description of data collection	Existing power market data set was extended by synthetically generating representative power network data. Data set for reliability analysis was generated by combining representative power system component parameters and reliability statistics based on historical data.
Data source location	The data set is compiled by combining multiple sources of primary data as summarized below and described in detail in the *Experimental Design, Materials, and Methods* section. Primary data source for reliability data: Data for the Norwegian Transmission System Operator (TSO) compiled from statistics collected through the Norwegian standardised system FASIT for collection, calculation and reporting of disturbance and reliability data. Primary data source for operating state data: Load demand measurements in Norway. Primary data source for network model: The network model was inspired by regions of the Nordic power system and generated using power system component data representative for the Norwegian power system.
Data accessibility	Repository name: Four-area test network DOI: https://doi.org/10.5281/zenodo.3923916 The raw disturbance and outage data underlying the reliability data set are confidential.
Related research article	I. B. Sperstad, E. H. Solvang, and S. H. Jakobsen, ‘A graph-based modeling framework for vulnerability analysis of critical sequences of events in power systems’, *International Journal of Electrical Power & Energy Systems*, vol. 125, 106408, 2021. DOI: https://doi.org/10.1016/j.ijepes.2020.106408.

## Value of the Data

• The data set is a complete and consistent test data set for power system reliability analyses: It comprises network data, including delivery point and generator information, with corresponding data for multiple operating states (load/generation composition), time-dependent reliability data (failure rates and outage times for network components), and basic interruption cost data.
• The power system model is representative for a part of the Nordic power system but has relatively limited size and accordingly low computation time, making it suitable for computationally intensive contingency analyses.
• Four distinct market (price) areas in the power system model makes it well suited for integrating multi-area power market models with reliability analyses. (The value of the data set is already proven through application in a series of case studies on integrated power system market and reliability analysis [1], [2], [3], [4], [5], [6], [7], [8], [9]. However, the data set was not made openly available in connection with these previous publications.) Multiple distinct areas also make the data set useful for studies of power system islanding.
• The data set can be of benefit to power system researchers and educational institutions for testing and benchmarking methods on a complete data set of relatively limited size.

## 1 Data Description

**Fig. 1:**
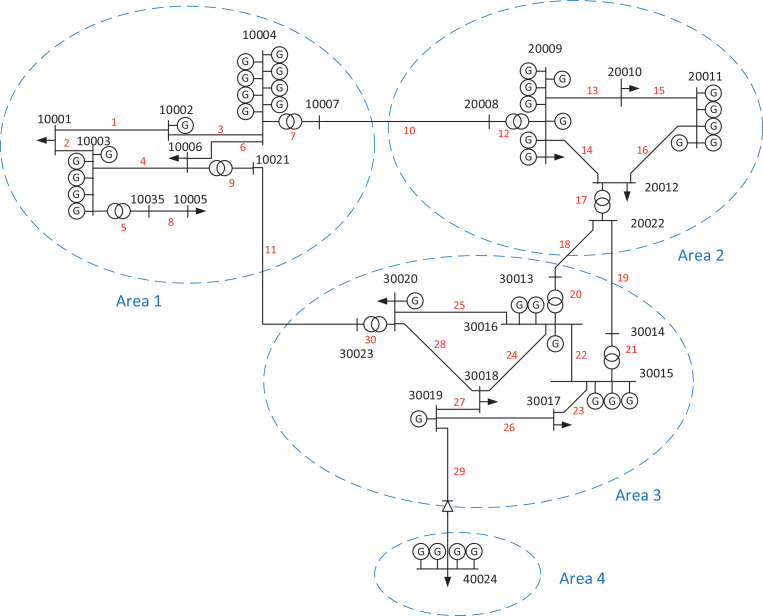
Four-area network with bus numbers, branches, generators and market areas (based on [10]).

This article presents a test data set combining data necessary for power system contingency and reliability analysis, including network data, reliability data, and operating state data (i.e. load/generation composition). The network model consists of 25 buses and four price (market) areas. Areas 1, 2 and 3 are meshed with a voltage level of 66 kV. These three areas are relatively weakly connected through 130 kV AC lines. The fourth area is represented by a single (import/export) bus connected by an HVDC cable to area 3. The single-line diagram for the four-area network model is shown in Fig. 1 along with bus numbers, branches, generators and market areas.

The data set consists of multiple files containing the following types of information:

• Network data: System buses, branches, and generator data (available both as .csv files on the MATPOWER case format [11] and a PSS®E .sav file [12])
• Operating state and delivery point data: Exemplary load and generation data for multiple operating states (.csv files) together with information on the delivery points
• Reliability data: Failure rate and outage time data for branches with scaling factors depending on the time of year (.csv files)

A brief description of the data files is given in Table 1.

**Table 1:** Overview of data files in the data set.

File name	Description of data
*4area_network_bus.csv*	Bus data on the MATPOWER case format [11]
*4area_network_gen.csv*	Generator data on the MATPOWER case format [11]
*4area_network_branch.csv*	Branch data on the MATPOWER case format [11]
*4area_network_gencost.csv*	Generation cost data on the MATPOWER case format [11]
*4area_network_areas.csv*	Market/price areas data on the MATPOWER case format [11]
*4area_network.sav*	Network data on the PSS®E case file format [12]
*4area_network_loaddata.csv*	Delivery point data with load demand and interruption cost data on the format described in more detail below (*operating state and delivery point data*)
*4area_network_gendata.csv*	Generation data on the format described in more detail below (*operating state and delivery point data*)
*4area_network_opstates.csv*	Specification of the time and duration of each operating state on a format described in more detail below (*operating state and delivery point data*)
*custdata_relative_load.csv*	Time-dependent variations in load demand on a format described in more detail below (*operating state and delivery point data*)
*4area_network_outagedata.csv*	Reliability data (for network components excluding protection and control equipment) on a format described in more detail below (*reliability data*)
*4area_network_protcontdata.csv*	Reliability data for protection and control equipment on a format described in more detail below (*reliability data*)
*compdata_component_relative_lambda.csv*	Time-dependent variations in failure rates on a format described in more detail below (*reliability data*)
*compdata_component_relative_outage.csv*	Time-dependent variations in outage times on a format described in more detail below (*reliability data*)

### 1.1 Network data

The single-line diagram in Fig. 1 gives a graphical depiction of the network topology (*4area_network_branch.csv*) as well as the generators (*4area_network_gendata.csv*) and delivery points (*4area_network_loaddata.csv*) in the system. Table 2 gives an overview of the generation and load demand in the system. The system is dominated by hydropower generation (in areas 1–3), whereas the generators in area 4 represent thermal power plants and wind power plants. (The first of the four generators in area 4 in *4area_network_gendata.csv* represents wind power and the three other area 4 generators represent thermal power plants. The generation and load demand data summarized in Table 2 are described in more detail in the data files *4area_network_gen.csv* and *4area_network_loaddata.csv*.) Bus 30,019 is modelled as the swing bus in the network model. The baseMVA value of the network model is 100.

**Table 2:** Overview of generation and load demand for each area.

Generation/load data	Area 1	Area 2	Area 3	Area 4	
Type of generation	Hydro	Hydro	Hydro	Thermal	Wind
Number of generators	21	12	8	3	1
Generation capacity (MW)	610	533	820	230	119
Peak load demand (MW)	582	641	588	68	

The network model exists in several variants that have been created to investigate grid investment decisions and the effects of higher levels of meshing within the areas and the ability of areas to operate independently. An overview of the variants is given in Table 3.

**Table 3:** Overview of variants of the network model.

Network model variant	References
Base version	[1, 2, 3, 4, 5, 6]
Without generation in area 4	[8, 10]
Without branch 25	[13, 9]
Without branch 23 and 25	[9]
Without branch 25 and without generation in area 4	[7]

### 1.2 Operating state and delivery point data

The data set includes 12 operating states (OSs). The operating states represent the load and generation composition in the network 10 a.m. on a Monday for one month in the year each. Operating states 1–12 correspond to months January–December. The time and duration of each operating state is specified in the file *4area_network_opstates.csv*. Data for load demand and generation in the operating states are contained in *4area_network_loaddata.csv and 4area_network_gendata.csv*, respectively.

The format for the load data file (*4area_network_loaddata.csv*) is shown in Table 4. Here, there is one row for each delivery point (load bus). The three first data columns are for bus numbers, customer type for the customers at this delivery point (e.g. industry or commercial) and average interruption costs for customers connected to the bus, respectively. This interruption cost can be used to specify the order in which to shed bus loads for each bus in an Optimal Power Flow-based model for load shedding [8]. These exemplary interruption cost data can also be used in the reliability analysis to calculate expected interruption costs if customer damage functions and other detailed interruption cost data are not included. The remaining columns describe the demand of each delivery point for all 12 operating states included in the data set. The values for the load demand are given in MW. An extract of load demand data for multiple operating states is given in Table 4. This extract includes two delivery points (load buses 10,001 and 10,005) and specifies the load demand for two operating states 1 and 2.

**Table 4:** Example load demand data for multiple operating states (*4area_network_loaddata.csv*).

	Bus no	Customer type	Interruption cost (NOK/kWh)	Load demand	Load demand	…
OS no				1	2	…
	10,001	1	220.3	258.8	257.8	…
	10,005	1	220.3	0	0	…
	…		…	…	…	…

The data set also includes data on the relative variation in load demand depending on the month of the year, day of the week, and hour of the day for each customer type. These time-dependent scaling factors are included in the data file *custdata_relative_load.csv* on a format described in Table 5.

**Table 5:** Time-dependent scaling factors for load demand (from *custdata_relative_load.csv*).

	1: Commercial	2: Industry	…
January	1.1701	1.1173	…
February	1.1654	1.113	…
…	…	…	…
Monday	1.1038	1.2102	…
Tuesday	1.1038	1.2102	…
…	…	…	…
hour 1	0.738	0.5716	…
hour 2	0.738	0.5716	…
…	…	…	…

The generation data file (*4area_network_gendata.csv*) is of a similar structure as the load data file (*4area_network_loaddata.csv*), with a column for bus numbers in addition to one column for each operating state. The generation values are given as the average active power output during the operating state and is measured in MW. Table 6 contains an extract with generation at buses 10,002 and 10,003 for operating states 1 and 2.

**Table 6:** Example generation data for multiple operating states (4area_network_gendata.csv).

	Bus no	Generation	Generation	…
OS no		1	2	…
	10,002	197.9	197.1	…
	10,003	47.5	47.3	…
	…	…	…	…

An extract of *4area_network_opstates.csv* is shown in Table 7. The time and duration of the operating states are specified by three columns each from row 3 downwards, and the number of the operating state is stated in row 2. The first of the three columns specify which months of the year (1–12) are included in the operating state, the second column specifies the days of the week (1–7) of these months that should be included, and the third column specifies which hours of the day (1–24) of these days and months that are included. In the example, operating state 1 is defined to cover every hour of every day in the January, etc.

**Table 7:** Example data on the time and duration of each operating state (*4area_network_opstates.csv*).

	Month	Day	Hour	Month	Day	Hour	Month	Day	…
OS no	1	1	1	2	2	2	3	3	…
	1	1	1	2	1	1	3	1	…
		2	2		2	2		2	…
		3	3		3	3		3	…
		…	…		…	…		…	…

### 1.3 Reliability data

Reliability data for network components are described in *4area_network_outagedata.csv* (for network components excluding protection and control equipment) and in *4area_network_protcontdata.csv* for protection and control equipment. The files describe one component per row and has data on what type of equipment a component is and on what its reliability data are. Note that each row of *4area_network_protcontdata.csv* represents a circuit breaker on the "from bus" end of the branch, so here there can be two rows per branch. An extract of *4area_network_outagedata.csv* and *4area_network_protcontdata.csv* is given in Table 9 and Table 10, respectively. A description of each column is given in Table 8. The component categories defined by the value in the Type column are described in Table 13 (in Section *Experimental Design, Materials, and Methods*).

**Table 8:** Reliability data explanations (for *4area_network_outagedata.csv* and *4area_network_protcontdata.csv*).

Column	Description
#	ID of the branch
*Main Type*	Which main type of component the branch represents (value 1 for transmission line, value 3 for transformer, 4 for protection and control equipment)
*Type*	Which type of component, defined by voltage levels and power ratings
*From bus*	The from bus terminal of the branch
*To bus*	The to bus terminal of the branch
*lambda*	Failure rate given as the expected number of failures per year (for *4area_network_protcontdata.csv*, the failure rate refers to unwanted spontaneous tripping of circuit breaker, i.e. fault type 2 in [7, 14])
*r*	Outage time (expected value) in hours
*length*	Length of the power line in kilometres
*Pm*	Conditional probability of missing tripping of circuit breaker (fault type 3 in [7, 14])
*Pu*	Conditional probability of unwanted tripping of circuit breaker (fault type 4 in [7, 14])
*rtime*	Time to restore supply after missing or unwanted tripping of breaker

Further, there is data on time-dependent scaling factors for failure rates and outage times in *compdata_component_relative_lambda.csv* and *compdata_component_relative_outage.csv*, respectively*.* The scaling factors in these files can be multiplied with the failure rate or outage time, respectively, of individual components. By doing so, one can e.g. get the failure rate at any month of the year, day of the week or hour of the day. An extract of the failure rate time-dependence data is given in Table 11 for components of type 1 and 2 and for the first two months of the year, first two days of the week and first two hours of a day. Note that although failure rates are constant within each month for the component types in Table 11, this is not the case in general. The data format for the time dependence of outage times (*compdata_component_relative_outage.csv*) is the same as for failure rates, and can be similarly applied.

## 2 Experimental Design, Materials and Methods

**Fig. 2:**
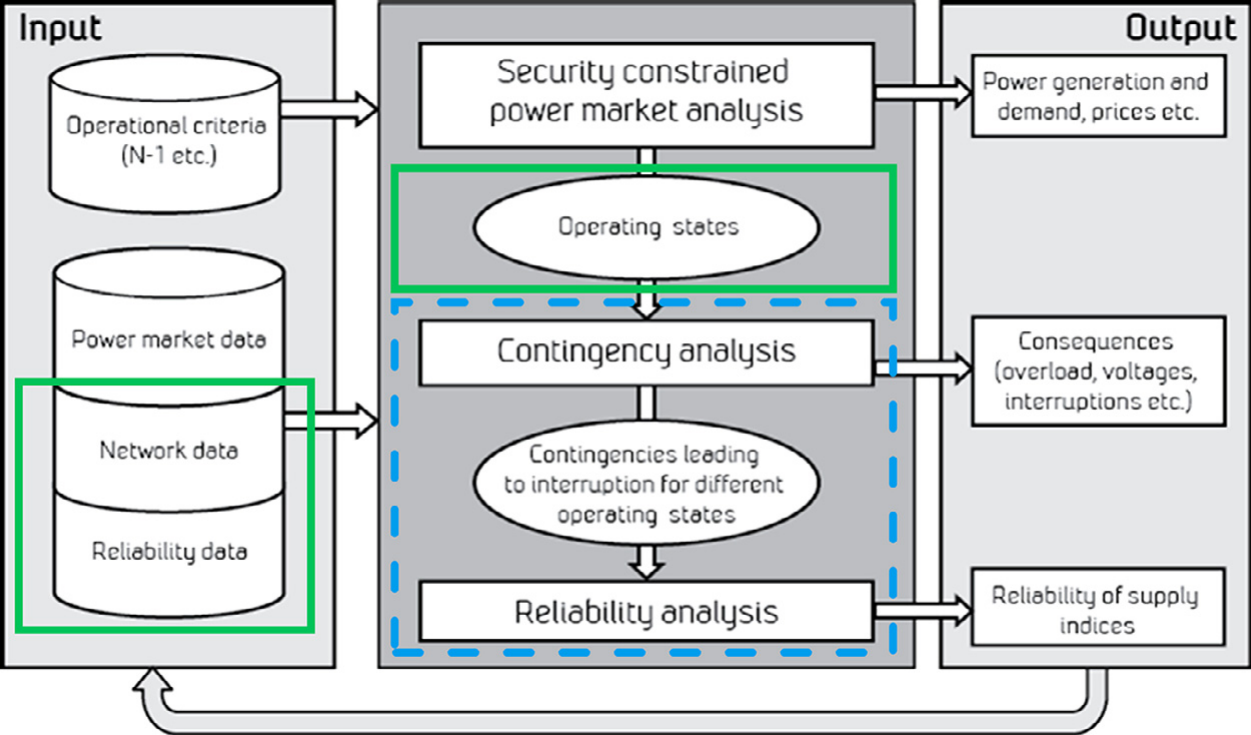
Framework for integrated power market and reliability of supply analysis [9], with the scope of the analysis and data set considered in this article indicated by blue and green rectangles, respectively.

The data set was designed for demonstrating the integration of a power market analysis for providing representative operating states to a reliability of supply analysis [7,9,15]. The integration was done according to the general SAMREL framework for integrated reliability of supply analysis illustrated in Fig. 2. This framework integrates a power market analysis, a contingency analysis and a reliability analysis. The input data required for the full analysis chain is indicated on the left-hand side of the figure. As explained in more detail below, the data set presented in this article refers only to the contingency and reliability analysis part of the analysis chain, as indicated in Fig. 2 by a dashed blue rectangle. With this scope for the analysis, the input data that is required are indicated by green rectangles in the figure. The green rectangles thus define the set of data that were needed for the consistent data set presented in this article.

### 2.1 Network Data

The test network first described in [9,13] originates from a previous test data set for multi-area power market models with network constraints between the areas but without any representation of the network within each area [16]. The original power market data set included data for three real watercourses in the hydropower-dominated Norwegian power system for each of the areas 1–3. Area 4 was later added to the system to represent import/export for the connection to a power market area with a large share of wind power generation.

Network data for real power systems in Norway are generally defined as sensitive information and is subject to confidentiality. A synthetic network model therefore had to be created and adapted to the power market data set to test and benchmark power market models that include a detailed network representation in the analysis, as presented in [13]. The network model was designed to be relatively strongly meshed networks within each area and weaker connections with longer lines between the areas. Generators and generation capacity was furthermore matched to the power plants of the power market data set. Representative parameter values for 66 kV and 130 kV transmission lines and transformers in the Norwegian power system were chosen for the branches in the network model. For simplicity, the HVDC cable between area 3 and 4 is in the network model represented by a 66 kV AC connection of corresponding capacity.

### 2.2 Operating state and delivery point data

Since the detailed power market data underlying this test data set and the relevant market models are not openly available, an exemplary set of operating states is included to form a complete test data set for contingency analysis and reliability analysis. 12 representative operating states were generated by taking an existing base case operating state generated by the market model [13] and scaling the load demand for each delivery point by time-dependent scaling factors for the corresponding customer type. This time dependence given in Table 5 is based on a Norwegian load demand data set, with measurements dating back to the early 1990′s, that has previously been generated and used by SINTEF Energy Research.

The six customer types defined in the Norwegian Cost of Energy Not Supplied (CENS) scheme are listed in Table 12. The scope of the data set presented in this article does not include customer damage functions and time-dependent interruption costs, and for more information on the CENS scheme and interruption cost data relevant for the Norwegian power system we refer to [17]. Table 12 also includes the Norwegian type descriptions found in [17]. In addition, simplified (not time-dependent) interruption cost data measured in NOK (cost level 2017) per kWh of energy not supplied are included in the rightmost column of Table 12. These data are obtained by calculating the interruption costs for an interruption with duration 1 hour at the reference time using the interruption cost data in [17] (the Norwegian CENS rates valid from 2019). In the data specified for the delivery points of the test network in Table 4, the interruption cost data in Table 12 were used.

### 2.3 Reliability data

The reliability data are primarily based on the Norwegian standardised system FASIT for collection, calculation and reporting of disturbance and reliability data [18]. This system is used to derive expected values for power system component reliability parameters such as failure rates per line length, outage time and more for the Norwegian power system. The FASIT data used for this dataset are based on statistics from the Norwegian TSO Statnett covering the period 1996–2005, and only permanent faults are included [19]. Aggregated reliability data for different component types are given in Table 13 below. The raw disturbance and outage data underlying the reliability data set are confidential. The protection and control equipment reliability data in Table 10 are the same representative input data as stated in [14,20].

In Table 13, the parameter *lambda* is the expected failure rate per year per kilometer line length, and *r* is the expected outage time in hours. These expected values were applied to the grid model so that failure rates and outage times of the network components as shown in Table 9 are representative for the Norwegian power system. The failure rate of each branch in the network was obtained by multiplying the *lambda* value in Table 13 by the *length* value for the branch. Component type number 18 in Table 13 is a test component used to demonstrate reliability data with dependence on the hour of the day and the day of the week, cf. Table 11 and [21]. These time dependence data are based on the data set prepared for [22], which in turn is based on reliability statistics for Statnett from the period January 1983 – June 1995. The monthly time dependence for the outage time has been smoothed so that the outage time is constant within each season.

**Table 9:** Extract of reliability data for the four-area network (from *4area_network_outagedata.csv*).

#	*Main Type*	*Type*	*From bus*	*To bus*	*lambda*	*r*	*length*
1	1	4	10,001	10,002	0.0045	16.7	1
2	1	4	10,001	10,003	0.009	16.7	2
…	…	…	…	…	…	…	…

**Table 10:** Extract of protection and control equipment reliability data for the four-area network (from *4area_network_protcontdata.csv*).

#	*Main Type*	*Type*	*From bus*	*To bus*	*lambda*	*r*	*Pm*	*Pu*	*rtime*
1	4	4	10,035	10,005	0.025	2	0.0205	0.007	0.5
2	4	4	10,001	10,002	0.025	2	0.0205	0.007	0.5
…	…	…	…	…	…	…	…

**Table 11:** Scaling factors for failure rates (from *compdata_component_relative_lambda.csv*).

	Type 1	Type 2	…
January	1.332	1.332	…
February	1.020	1.020	…
…	…	…	…
Monday	1	1	…
Tuesday	1	1	…
…	…	…	…
hour 1	1	1	…
hour 2	1	1	…
…	…	…	…

**Table 12:** Customer types according to the Norwegian Cost of Energy Not Supplied (CENS) scheme with exemplary (not time-dependent) interruption cost data.

Type number	Customer type	Customer type (type description in Norwegian)	Interruption cost [NOK/kWh]
1	Commercial	Handel og tjenester	220.3
2	Industry	Industri	132.6
3	Residential	Husholdning	23.5
4	Agriculture	Jordbruk	21.4
5	Public service	Offentlig virksomhet	194.5
6	Energy-intensive Industry	Industri med eldrevne prosesser	58.2

**Table 13:** Component types with reliability data.

Component type	*Main type*	*Type*	*lambda*	*r*
Overhead transmission line 420 kV	1	1	0.0008	28.5333
Overhead transmission line 300 - 220 kV	1	2	0.0007	61.5833
Overhead transmission line 132 kV	1	3	0.0019	91.4000
Overhead transmission line 110 - 33 kV	1	4	0.0045	16.7000
Power cable 420 kV	1	5	0.0000	0.0000
Power cable 330 - 220 kV	1	6	0.0030	14.8000
Power cable 132 kV	1	7	0.0174	374.7167
Power cable 110 - 33 kV	1	8	0.0093	101.2167
Transformer 420 kV	3	9	0.0083	536.8000
Transformer 300 - 220 kV	3	10	0.0122	1000.2000
Transformer 132 kV	3	11	0.0036	367.6333
Transformer 110 - 33 kV	3	12	0.0057	30.8000
Generator 420 - 132 kV, > 150 MVA	5	13	0.5665	20.8833
Generator 420 - 132 kV, 150 - 100 MVA	5	14	0.1836	60.0500
Generator 420 - 132 kV, 100 - 50 MVA	5	15	0.1611	124.8833
Generator 420 - 132 kV, < 50 MVA	5	16	0.1150	70.3333
Generator 110 - 33 kV, 0 - 120 MVA	5	17	0.0988	70.3333
Overhead transmission line (example component, 300–420 kV)	1	18	N/A	N/A

## CRediT Author Statement

**Iver Bakken Sperstad:** Conceptualization, Data curation, Project administration, Software, Writing - original draft. **Espen Hafstad Solvang:** Data curation, Writing - original draft, **Sigurd Hofsmo Jakobsen:** Data curation, Software, Writing - original draft. **Oddbjørn Gjerde:** Conceptualization, Data curation, Funding acquisition, Investigation, Methodology, Writing - review & editing.

## Declaration of Competing Interest

The authors declare that they have no known competing financial interests or personal relationships which have, or could be perceived to have, influenced the work reported in this article.
